# Characterization of the multigene family *TaHKT 2;1* in bread wheat and the role of gene members in plant Na^+^ and K^+^ status

**DOI:** 10.1186/1471-2229-14-159

**Published:** 2014-06-11

**Authors:** HA Chandima K Ariyarathna, Tanveer Ul-Haq, Timothy D Colmer, Michael G Francki

**Affiliations:** 1 School of Plant Biology and Institute of Agriculture, The University of Western Australia, Crawley 6009, Western Australia; 2 State Agricultural Biotechnology Centre, Murdoch University, Murdoch 6150, Western Australia; 3 College of Agriculture, D. G. Khan, University of Agriculture Faisalabad, Faisalabad 38040, Pakistan; 4 Department of Agriculture and Food Western Australia, South Perth 6151, Western Australia

**Keywords:** IWGSS, *Cis* regulatory elements, Gene expression, Aneuploid lines, Tissue Na^+^, Tissue K^+^

## Abstract

**Background:**

A member of the *TaHKT2;1* multigene family was previously identified as a Na^+^ transporter with a possible role in root Na^+^ uptake. In the present study, the existing full-length cDNA of this member was used as a basis to query the International Wheat Genome Survey Sequence to identify all members of the *TaHKT2;1* family. Individual *TaHKT2;1* genes were subsequently studied for gene and predicted protein structures, promoter variability, tissue expression and their role in Na^+^ and K^+^ status of wheat.

**Results:**

Six *TaHKT2;1* genes were characterized which included four functional genes (*TaHKT2;1 7AL-1, TaHKT2;1 7BL-1, TaHKT2;1 7BL-2* and *TaHKT2;1 7DL-1*) and two pseudogenes (*TaHKT2;1 7AL-2* and *TaHKT2;1 7AL-3*), on chromosomes 7A, 7B and 7D of hexaploid wheat. Variability in protein domains for cation specificity and in *cis*-regulatory elements for salt response in gene promoters, were identified amongst the functional *TaHKT2;1* members. The functional genes were expressed under low and high NaCl conditions in roots and leaf sheaths, but were down regulated in leaf blades. Alternative splicing events were evident in *TaHKT2;1 7AL-1*. Aneuploid lines null for each functional gene were grown in high NaCl nutrient solution culture to identify potential role of each *TaHKT2;1* member. Aneuploid lines null for *TaHKT2;1 7AL-1*, *TaHKT2;1 7BL-1* and *TaHKT2;1 7BL*-2 showed no difference in Na^+^ concentration between Chinese Spring except for higher Na^+^ in sheaths. The same aneuploid lines had lower K^+^ in roots, sheath and youngest fully expanded leaf but only under high (200 mM) NaCl in the external solution. There was no difference in Na^+^ or K^+^ concentration for any treatment between aneuploid line null for the *TaHKT2;1 7DL-1* gene and Chinese Spring.

**Conclusions:**

*TaHKT2;1* is a complex family consisting of pseudogenes and functional members. *TaHKT2;1* genes do not have an apparent role in controlling root Na^+^ uptake in bread wheat seedlings under experimental conditions in this study, contrary to existing hypotheses. However, *TaHKT2;1* genes or, indeed other genes in the same chromosome region on 7AL, are candidates that may control Na^+^ transport from root to sheath and regulate K^+^ levels in different plant tissues.

## Background

Saline soils are a challenge to cereal production in many regions of the world. Osmotic and ion-specific damage that significantly reduces crop growth in saline soils is largely due to excess of Na^+^ and Cl^−^ ions [1,2]. In bread wheat, a balance between restricting Na^+^ net uptake at roots and subsequent transport into shoots and sub-cellular compartmentation to cope with unfavourable levels of Na^+^ is needed to maintain ion homeostasis for plant growth and development under saline conditions [3,4]. The regulation of Na^+^ transport and its genetic basis is, therefore, of significant interest in order to assist in development of targeted strategies for enhanced salinity tolerance in crops.

A major portion of Na^+^ exclusion (>98%) in bread wheat is accomplished by restricting net Na^+^ uptake at the soil-root interface and net xylem loading in roots [2], yet a number of channels and transporters facilitate Na^+^ influx when Na^+^ concentration is high in the soil solution [5-8]. Channel and transporter proteins are potential targets to reduce Na^+^ net uptake by roots and have been a major focus to investigate their role in crop tolerance to salinity. Electrophysiological observations and pharmacology of ^22^Na^+^ tracer influxes in cereal roots support the hypothesis that the bulk of Na^+^ influx is mediated by voltage-independent, non-selective cation channels (VICs/NSCCs) [9-11]. In addition, two transporters *LCT1*[12,13] and *HKT2;1*[14] can mediate high and/or low-affinity Na^+^ transport in wheat. While both VICs/NSCCs [15] and *LCT1*[13] facilitate Ca^2+^ dependant unidirectional influx of various cations, *TaHKT2;1* mediates Ca^2+^ independent influx of Na^+^ and K^+^[16,17]. Although, *TaHKT2;1* is a Na^+^/K^+^ co-transporter, the protein has a minor significance in K^+^ nutrition as Na^+^ - coupled K^+^ uptake has limited physiological relevance in terrestrial species [18]. Therefore, among the genes encoding candidate proteins involved in root Na^+^ influx, *TaHKT2;1* is of particular interest in crop breeding because of its high specificity for Na^+^ and the potential to manipulate *TaHKT2;1* genes without largely interfering with the homeostasis of nutritionally important ions (e.g., K^+^, Ca^2+^) in plants.

*TaHKT2;1* has a Gly-Gly-Gly-Gly type K^+^/Na^+^ selectivity pore [19,20] and when expressed in *Xenopus* oocytes and yeast heterologous systems, preferentially mediates high affinity K^+^ uptake when external Na^+^ is at sub-millimolar levels, but facilitates low-affinity Na^+^ influx when millimolar levels of external Na^+^ are in excess of K^+^[16,21,22]. The ion transport properties of *TaHKT2;1* were determined when point mutations within the *TaHKT2;1* gene improved K^+^ specificity and reduced Na^+^ influx in *Xenopus* oocytes and yeast [21,23]. Down regulation of *TaHKT2;1* in transgenic bread wheat by expressing anti-sense or truncated sense constructs reduced Na^+^ levels in K^+^ starved seedling roots [24]. Since the gene is expressed in the cortical cells of roots, alongside in xylem parenchyma in leaves, it is possible that the protein encoded by *TaHKT2;1* is associated with root Na^+^ uptake and translocation [24]. Therefore, it appears that under saline conditions *TaHKT2;1* is responsible for substantial Na^+^ influx into roots of bread wheat and transport to other parts of the plant.

*TaHKT2;1* has been identified as a multi-gene family consisting of five individual members distributed across homeologous chromosome group 7 in bread wheat [25]. Although previous studies identified the function of Na^+^ transport in one member of the *TaHKT2;1* gene family in heterologous systems [21,23] and in transgenic plants by down-regulation of one or more genes [24], the contribution of each member of the gene family and their interactions to control ion accumulation in wheat remains unknown. This study aimed to understand the organization and variability of each member of the *TaHKT2;1* gene family, transcriptional regulation, their encoded proteins, and potential contributions to controlling Na^+^ and K^+^ status in bread wheat. The study used an existing full length cDNA (FL-cDNA) sequence (U16709) as a basis to select and characterize all members of the *TaHKT2;1* gene family using the International Wheat Genome Survey Sequence (IWGSS) (http://www.wheatgenome.org).

## Results

### Organization and structure of *TaHKT2;1* multi-gene family

**Figure 1:**
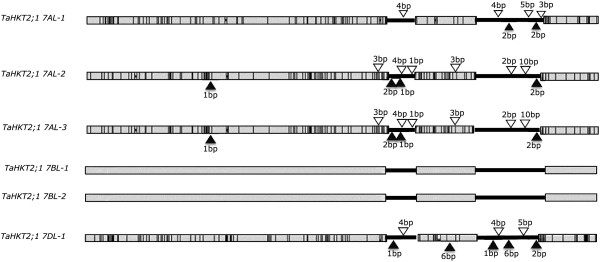
**Gene structure of each member of the *TaHKT2;1 *gene family.** Exons are shown in grey boxes interrupted by 2 introns (black bars). Sub-genome specific sequence variations were identified in reference to *TaHKT2;1 7BL-1/-2*. Black vertical bars indicate SNPs, and open triangles and close triangles indicate insertions and deletions, respectively.

The FL-cDNA of *TaHKT2;1*, U16709, was used as query sequence in BLASTN analysis of IWGSS to identify members of the *TaHKT2;1* gene family in hexaploid wheat. Six significant hits having greater than 90% nucleotide identity with the FL-cDNA were retrieved that identified three *TaHKT2;1* genes on the long arm of chromosome 7A (*TaHKT2;1 7AL-1*, *TaHKT2;1 7AL-2*, *TaHKT2;1 7AL-3*), two genes on the long arm of 7B (*TaHKT2;1 7BL-1*, *TaHKT2;1 7BL-2*), and one on the long arm of 7D (*TaHKT2;1 7DL-1*). The six genes were further studied for gene structure and sequence variability. Gene structure was analysed by splice junction prediction software, and subsequently validated in DNA sequence alignments with the FL-cDNA, U16709, revealed three exons interrupted by two introns for each gene (Figure 1) with intron splice junction sites having the conserved motif, 5′ GT- AG 3′. *TaHKT2;1 7AL-2* and *TaHKT2;1 7AL-3* were identical in their introns and exons and each had a single nucleotide deletion at +191 bp in exon 1 relative to the other genes on chromosome 7A, 7B and 7D whereby the translated sequences predicted an in-frame stop codon causing pre-mature termination and a significantly truncated protein of 65 amino acid residues. Therefore, it appeared that *TaHKT2;1 7AL-2* and *TaHKT2;1 7AL-3* were duplicated pseudogenes. Accordingly, *TaHKT2;1* represented a multi-gene family of six individual genes in bread wheat of which four (*TaHKT2;1 7AL-1*, *TaHKT2;1 7BL-1*, *TaHKT2;1 7BL-2* and *TaHKT2;1 7DL-1*) were identified as functional members.

The functional *TaHKT2;1* genes were further analysed using multiple DNA sequence alignments and revealed 90-94% nucleotide identity (Table 1). The two genes, *TaHKT2;1 7BL-1* and *TaHKT2;1 7BL-2* were identical in introns and exons (thus hereafter designated as *TaHKT2;1 7BL-1/-2*) and shared 100% nucleotide identity with the full-length cDNA, U16709, so it was reasonable to assume that these genes on 7BL were the source of the query cDNA sequence. *TaHKT2;1 7BL-1/-2* were subsequently used in pairwise sequence comparisons with *TaHKT2;1 7AL-1* and *TaHKT2;1 7DL-1* to identify variations in intron and exon regions (Figure 1 and Table 2). Sequence divergence in the intron regions of the *TaHKT2;1* genes were clearly evident, including SNPs and INDELs (Table 3). Exon sequence variation between *TaHKT2;1 7BL-1/-2* and genes on 7A and 7D were largely represented by SNPs, whereby 36% of the SNPs in *TaHKT2;1 7AL-1* and 41% of the SNPs in *TaHKT2;1 7DL-1* represented non-synonymous substitutions (Table 3). Thus, sequence comparisons against *TaHKT2;1 7BL-1/-2* identified members of *TaHKT2;1* gene family exhibiting a degree of exon variability resulting in predicted protein differences.

**Table 1:** **Percent DNA sequence and amino acid (parentheses) identity between functional members of the *TaHKT2;1 *** **gene family**

**Gene**	***TaHKT2;1 7AL-1***	***TaHKT2;1 7BL-1***	***TaHKT2;1 7DL-1***
		***TaHKT2;1 7BL-2***	
*TaHKT2;1 7AL-1*	-		
*TaHKT2;1 7BL-1* and *TaHKT2;1 7BL-2*	91.3 (92.5)	-	
*TaHKT2;1 7DL-1*	94.3 (93.4)	90.1 (91.6)	-

**Table 2:** **Attributes of exons, introns and proteins of functional members of the *TaHKT2;1 *** **gene family**

		***TaHKT2;1 7AL-1***	***TaHKT2;1 7BL-1 TaHKT2;1 7BL-2***	***TaHKT2;1 7DL-1***
Exon	Exon 1 length (bp)	1172	1172	1172
	Exon 2 length (bp)	228	228	222
	Exon 3 length (bp)	202	202	202
	Non-synonymous SNPs	35	-	43
	Total number of SNPs	95		104
	Insertions (bp)	0	-	0
	Deletions (bp)	0	-	6
Intron	Intron 1 length (bp)	130	126	129
	Intron 2 length (bp)	263	256	256
	SNPs	59	-	61
	Insertions (bp)	16	-	13
	Deletions (bp)	4	-	10
Protein	Amino acid length	533	533	531
	Amino acid substitutions	31	-	35

SNPs and INDELS were identified using *TaHKT2;1 7BL-1/ TaHKT2;1 7BL-2* as the reference sequence*.*

**Table 3:** **Salt induced *cis– *** **acting regulatory elements identified in the promoter regions of the functional *TaHKT2;1 *** **genes**

***Cis*-regulatory element**	**Conserved sequence motif**	**Gene**	**Associated families of salt-stress activated TFs**	**Reference**
ABRE	ACGTGG/T	*TaHKT2;1 7AL-1*(1)	AREB/ABF (ABA responsive element- binding proteins)	[26]
		*TaHKT2;1 7BL-2*(1) *TaHKT2;1 7DL-1*(5)		
AtMYC2	CACATG	*TaHKT2;1 7AL-1*(1) *TaHKT2;1 7BL-1*(2) *TaHKT2;1 7BL-2*(3) *TaHKT2;1 7DL-1*(5)	MYC/MYB (myelocytomatosis/myeloblastosis)	[27]
DRE/CRT	CCCGAC	*TaHKT2;1 7AL-1*(1) *TaHKT2;1 7BL-1*(2) *TaHKT2;1 7BL-2*(1) *TaHKT2;1 7DL-1*(1)	CBF/DREB (C-repeat binding factor/dehydration- responsive element-binding proteins)	[28]
MYCATERD1	CATGTG	*TaHKT2;1 7AL-1*(3) *TaHKT2;1 7BL-1*(2) *TaHKT2;1 7BL-2*(3) *TaHKT2;1 7DL-1*(4)	plant specific family of TFs, NAC (No apical meristem)	[29]
GT-1 box	GAAAAA	*TaHKT2;1 7BL-1*(10) *TaHKT2;1 7BL-2*(4) *TaHKT2;1 7DL-1*(12)	Trihelix family, also known as GT factors	[30]
W-Box	TTTGACY	*TaHKT2;1 7AL-1*(12) *TaHKT2;1 7BL-1*(10) *TaHKT2;1 7BL-2*(10) *TaHKT2;1 7DL-1*(10)	WRKY transcription factors	[31]

The number of each element in the promoter represented in each gene is shown in parenthesis.

### Analysis of predicted proteins of *TaHKT2;1* multi-gene family

**Figure 2:**
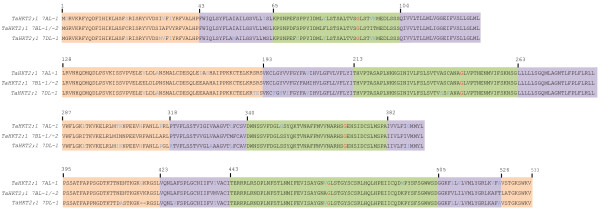
**Predicted proteins of functional members of the *TaHKT2;1 *gene family containing four membrane-pore-membrane structures.** Cytoplasmic, trans-membrane, and p-loop domains are in pink, purple and green backgrounds respectively. Amino acid substitutions are indicated in blue and the filter glycine residues in red.

Functional members of *TaHKT2;1* gene family on 7AL and 7BL encoded proteins of 533 amino acids whereas the gene on 7DL encoded a protein of 531 amino acids (Table 2) having 91% to 93% protein identity (Table 1) with *TaHKT2;1 7BL-1/-2*. Sequence alignment (Figure 2) coupled with analysis of hydrophobicity (Additional file 1: Figure S1) and 3D structures of the predicted proteins revealed the topology of four sequentially arranged membrane-pore-membrane (MPM) domains in each protein (Figure 2). Glycine molecules at Gly91, Gly246, Gly370 and Gly473 that form the selectivity pore filters [19,20] were conserved in each protein (Figure 2) predicting that each functional member has potential to mediate both K^+^ and Na^+^ transport. Interestingly, several amino acid substitutions were identified in the close proximity of filter glycine residues on first, second and fourth MPM domains of *TaHKT2;1 7AL-1* and *TaHKT2;1 7DL-1* compared to *TaHKT2;1 7BL-1/-2* (Figure 2). In the first M1_a_P_a_M2_a_, Ile95 in *TaHKT2;1 7BL-1/-2* was substituted by Val, an amino acid having similar physical properties, in both *TaHKT2;1 7AL-1* and *TaHKT2;1 7DL-1*. The substitution Thr96 for another hydrophilic but larger amino acid Lys was shared in both *TaHKT2;1 7AL-1* and *TaHKT2;1 7DL-1*. The second M1_b_P_b_M2_b_ identified two substitutions, one at Ala240 for Ser, a more hydrophilic amino acid in the same size range and Cys242 for a large hydrophobic amino acid Phe in *TaHKT2;1 7DL-1*. The fourth M1_d_P_d_M2_d_ identified Ala472 in both *TaHKT2;1 7AL-1 *and *TaHKT2;1 7DL-1,* substituted for Val, an amino acid carrying large aliphatic hydrophilic side chains*.* Altered physical properties and steric hindrance caused by the amino acid substitutions could, therefore, potentially change structural properties of the filters and manifest modified protein functions for ion selectivity and transport.

### Promoter analysis of functional *TaHKT2;1* members

Promoter diversity was investigated in functional members of the *TaHKT2;1* gene family by analysis of 2000 bp region up-stream of the predicted translation start site. The core promoter element, TATAA box (−69 bp) was conserved in all functional members of the *TaHKT2;1* family. Although *TaHKT2;1 7BL-1/-2* were identical in exon and intron sequences, they shared only 42.7% DNA sequence identity between promoters. The promoter region of *TaHKT2;1 7AL-1*, and *TaHKT2;1 7DL-1* genes showed 65.8% and 43.5% DNA sequence identity, respectively, compared with *TaHKT2;1 7BL-1/-2*. Table 3 summarizes the number and type of *cis*-acting regulatory elements (CREs) identified in promoters of functional members of the *TaHKT2;1* family using the PLANTcare and PLACE databases. Promoters of all functional *TaHKT2;1* genes contained a number of CREs known to be involved in salt activated response in plants and each gene shared at least five CREs of different categories. Therefore, it appeared that salt stress-activated transcription factors (TFs) interact with CREs to regulate *TaHKT2;1* genes.

### Analysis of *TaHKT2;1* pseudogenes in *Triticum* species

**Figure 3:**
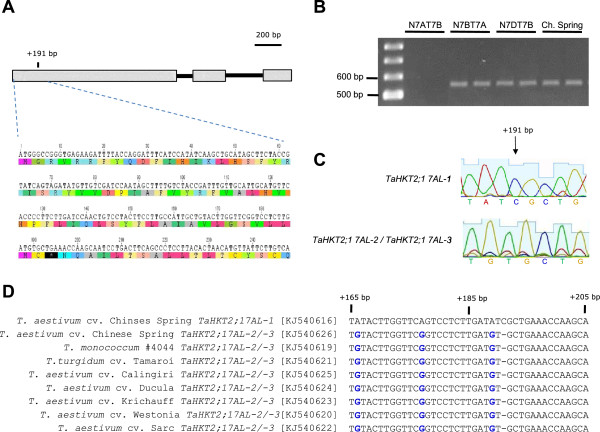
**Sequence analysis of single base deletion in pseudogenes *TaHKT2;1 7AL-2/ TaHKT2;1 7AL-3 *from different *Triticum *species*. *(A)** Gene structure and partial DNA sequence with predicted protein showing pre-mature stop codon indicated by an asterisk. **(B)** Nullisomic- tetrasomic analysis of gene specific primer pair 5′A2/3 F and 5′A2/3 R for amplifying and sequencing the region coding for premature stop codon. **(C)** Sequence chromatogram comparing the functional *TaHKT2;1 7AL-1* with *TaHKT2;1 7AL-2/TaHKT2;1 7AL-3* where the single base deletion is highlighted by an arrow. **(D)** Sequences of the functional gene *TaHKT2;1 7AL-1,* the conserved single base deletion at +191 bp in *TaHKT2;1 7AL-2/ TaHKT2;1 7AL-3* in Chinese Spring and in selected diploid, tetraploid and hexaploid Triticum species. Single base deletion is shown by dash whereas SNPs between accessions are indicated in blue.

The duplicated pseudogenes, *TaHKT2;1 7AL-2* and *TaHKT2;1 7AL-3*, showed an in-frame stop codon in exon 1 resulting in a truncated protein of 65 amino acids (Figure 3A) relative to its functional counterpart, *TaHKT2;1 7AL-1*. The pseudogenes were examined across selected *Triticum* species in order to gain further insight into their origin and evolution. The 5′ terminus of exon 1 including 50 bp upstream from the translation start site was amplified from diploid, tetraploid and hexaploid wheats using gene specific primers 5′A2/3 F and 5′A2/3 R (Table 4). Specificity of the primers that were used to amplify the region encoding the premature stop codon in exon 1 was confirmed by the absence of PCR amplicons from nullisomic-tetrasomic lines N7AT7B and N7AT7D (Figure 3B). The single base deletion at +191 bp in *TaHKT2;1 7AL-2/-3* relative to *TaHKT2;1 7AL-1* (Figure 3C) responsible for the in-frame stop codon was conserved in all diploid, tetraploid and hexaploid *Triticum* accessions analysed (Figure 3D) indicating that the deletion event was an ancient evolutionary sequence variant and the resulting pseudogenes were retained in modern durum and bread wheat accessions. Selection constraints on the genes were estimated by replacement (*Ka*) to silent (*Ks*) ratio [32]. The ratio between the functional genes *TaHKT2;1 7AL-1* and *TaHKT2;1 7BL-1/-2* was 0.20. Higher ratio value of 0.56 between *TaHKT2;1 7AL-2/ -3* and the functional counterpart *TaHKT2;1 7AL-1* indicated relaxed selection pressure on the pseudogenes.

**Table 4:** **Gene specific PCR primers targeting INDELs and SNPs to amplify individual members of the *TaHKT2;1 *** **gene family**

**Gene**	**Primer**	**Primer location (bp)**	**Primer sequence 5′-3′**	**PCR and the annealing temperature (°C)**
*TaHKT2;1 7AL-2* and *TaHKT2;1 7AL-3*	5′A2/3 F	−56	GAGACCTATCTTGACACGCAT	Touch down PCR at 55-50
*TaHKT2;1 7AL-2* and *TaHKT2;1 7AL-3*	5′A2/3 R	+489	GACTCATCACTTTGTGCTGC	Touch down PCR at 55-50
*TaHKT2;1 7AL-1*	A1 F	+1325	GCACCACCCAGTGATGAC	Touch down PCR at 55-50
*TaHKT2;1 7AL-1*	A1 R	+1894	CCAGAAAAGCTGTATCGCA	Touch down PCR at 55-50
*TaHKT2;1 7BL-1* and *TaHKT2;1 7BL-2*	B1/2 F	+1353	GAGAACACGAAAGGGAGAGT	Touch down PCR at 55-50
*TaHKT2;1 7BL-1* and *TaHKT2;1 7BL-2*	B1/2 R	+1933	TCCATAGAGCATGACCGAT	Touch down PCR at 55-50
*TaHKT2;1 7DL-1*	D F	+1357	CGAGCACAAAAGGCAAGA	Touch down PCR at 55-50
*TaHKT2;1 7DL-1*	D R	+1955	TGGACACCGCAAACTCT	Touch down PCR at 55-50
*TaHKT2;1 7AL-1*	FL:A1 F	−29	CATTTGTTTCTCCCAGTCG	Standard PCR at 53
*TaHKT2;1 7AL-1*	FL:A1 R	+2180	CCACACGTTGATAGATAATGTC	Standard PCR at 53
*TaHKT2;1 7BL-1* and *TaHKT2;1 7BL-2*	FL:B1/2 F	−19	CACACTCATACATAGCACCAT	Standard PCR at 53
*TaHKT2;1 7BL-1* and *TaHKT2;1 7BL-2*	FL:B1/2 R	+2251	TTCTCTTTCGCTACGATTGT	Standard PCR at 53
*TaHKT2;1 7DL-1*	FL:D F	−23	GTTTCTCACTCATATATAGGACCA	Standard PCR at 56
*TaHKT2;1 7DL-1*	FL:D R	+2039	GTACGCAGCAGTTATACCAG	Standard PCR at 56

### Expression of functional members of *TaHKT2;1* gene family

**Figure 4:**
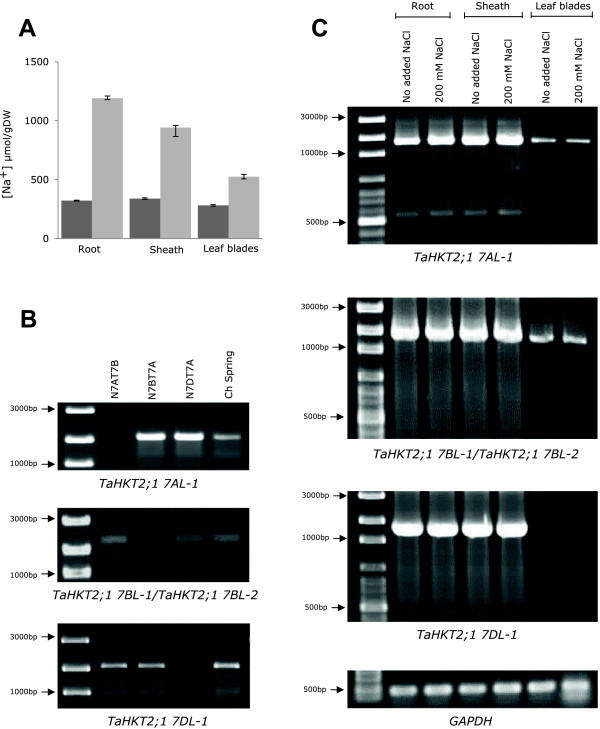
**Transcriptional analysis of *TaHKT2;1 *gene family in *T. aestivum *L. cv. Chinese Spring.** Tissue samples of each genotype were measured for ion concentration in triplicate and each genotype was replicated four times for each treatment. **(A)** Tissue Na^+^ concentrations in treatments with no added NaCl (dark grey) and 200 mM NaCl (light grey). **(B)** Nullisomic-tetrasomic analysis of gene specific primers used for transcript analysis. **(C)** RT-PCR and detection of FL- cDNA in roots, sheaths and leaf blades.

The expression of functional members of the *TaHKT2;1* gene family were investigated by imposing NaCl treatments on Chinese Spring seedlings and detection of transcripts specific for each gene in roots, sheaths and leaf blades. After three days exposure at 200 mM NaCl treatment in the external solution, Na^+^ concentration was 4-fold higher in roots, 3-fold in sheath and 2-fold in leaves, compared to the control plants with no added NaCl in the external solution (Figure 4A) and, therefore, showed significant (*P <* 0.01) phenotypic differences for transcript analysis of the different *TaHKT2;1* genes. Primers were designed to amplify FL-cDNA of each functional member of the *TaHKT2;1* gene family and gene specificity of primers was validated using nullisomic-tetrasomic lines (Figure 4B). *TaHKT2;1 7AL-1* and *TaHKT2;1 7BL-1/-2* were expressed in roots, sheaths and leaf blades under both low and high NaCl conditions whilst *TaHKT2;1 7DL-1* was identified only in roots and sheaths (Figure 4C). Each functional member of the *TaHKT2;1* gene family was down regulated in the leaf blades (Figure 4C). A unique feature of the *TaHKT2;1 7AL-1* gene was an additional smaller transcript of approximately 600 bp in length identified only in root and sheath tissues under both NaCl treated and non-treated conditions (Figure 4C), indicating that alternative splicing was evident for this gene in these tissues. No RT-PCR-amplification of *TaHKT2;1 7AL-2* or *TaHKT2;1 7AL-3* was detected in any of the tissues analysed (data not shown), providing further evidence that these were, indeed, unprocessed pseudogenes.

FL-cDNA transcripts of functional *TaHKT2;1* genes expressed in root, sheath and leaf under low and high NaCl conditions were sequenced. The cDNA sequence for *TaHKT2;1 7AL-1* [Genbank: KJ540616]; *TaHKT2;1 7BL-1/-2* [Genbank: U16709] and *TaHKT2;1 7DL-1* [Genbank: KJ540618] were identical from each tissue and were in perfect agreement with the predictions of gene structure in Figure 1 when aligned against the cognate genomic DNA sequence. Sequence analysis identified a 571 bp alternatively spliced transcript [Genbank: KJ540617] from *TaHKT2;1 7AL-1* encoding a predicted protein of 133 amino acids. Alignment with genomic DNA identified a splice site within exon 1 resulting in the deleting of exon 2 and exon 3 and intervening introns relative to its larger cDNA counterpart [Genbank: KJ540616]. The alternatively spliced *TaHKT2;1* 7*AL-1* sequence had the dinucleotide motif 5′-AT and AC-3′ at the exon-intron splice junction different to the conserved 5′GT and AG-3′ splice junction motifs in all other transcripts.

### Tissue ion analyses of wheat aneuploid lines null for *TaHKT2;1* genes

**Figure 5:**
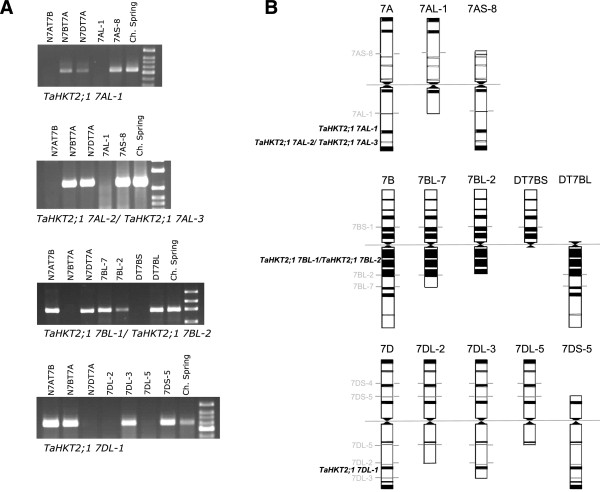
**Deletion bin mapping of the *TaHKT2;1 *gene family. (A)** Nullisomic tetrasomic and aneuploid line analysis using PCR primers specific for *TaHKT2;1 7AL-1, TaHKT2;1 7AL-2/TaHKT2;1 7AL-3, TaHKT2;1 7BL-1/TaHKT2;1 7BL-2* and *TaHKT2;1 7DL-1* genes. **(B)** Allocation of *TaHKT2;1* genes to deletion bin maps on homeologous group 7 chromosomes.

An investigation of possible phenotypic effects of *TaHKT2;1* genes was conducted by evaluating tissue Na^+^ and K^+^ concentrations in wheat aneuploid lines null for each member of the *TaHKT2;1* gene family, when exposed to NaCl and different K^+^ supply in the external solution. The absence of DNA amplicons from aneuploid lines using gene specific PCR primers (Figure 5A) confirmed that *TaHKT2;1 7AL-1,* and *TaHKT2;1 7DL-1* were located in the distal region of 7AL and 7DL respectively, whereas *TaHKT2;1 7BL-1/-2* were located within the proximal region of 7BL (Figure 5B)*.* Therefore, the aneuploid lines 7AL-1 and Dt7BS were null for the functional genes *TaHKT2;1 7AL-1* and *TaHKT2;1 7BL-1/-2* respectively, whereas 7DL-2 was null for *TaHKT2;1 7DL-1* and suitable for comparing ion concentrations with Chinese Spring under NaCl and K^+^ supply.

**Figure 6:**
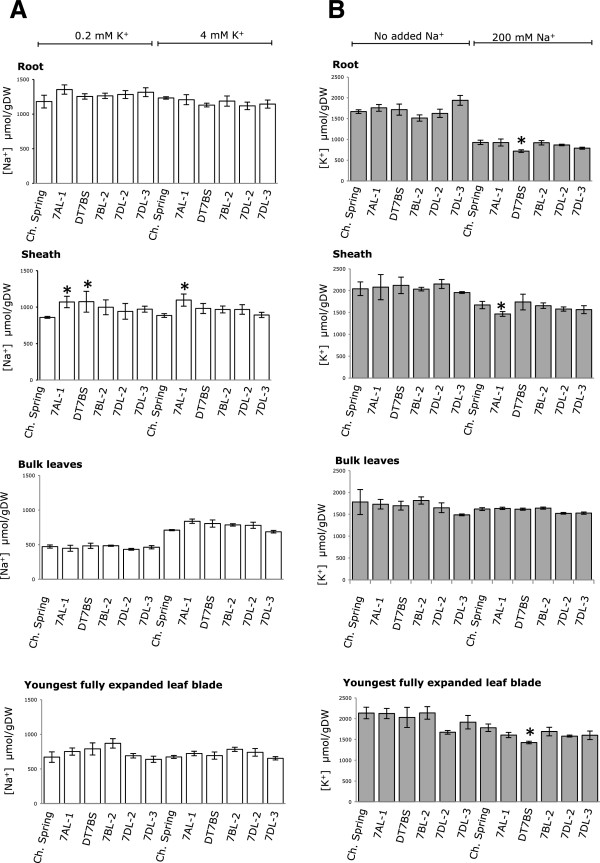
**Analysis of ion concentration in tissues from Chinese Spring and wheat aneuploid lines null for each functional *TaHKT2;1 *genes.** Tissue samples of each genotype were measured for ion concentration in triplicate and each genotype was replicated four times for each salt treatment. **(A)** Mean Na^+^ concentrations (±SE) of various tissues of each genotype subjected to 200 mM NaCl treatment in the presence of 0.2 mM or 4 mM K^+^. **(B)** Mean K^+^ concentrations (±SE) of various tissues of each genotype subjected to 4 mM K^+^ treatment in no added Na^+^ in or the presence of 200 mM NaCl. Significant differences in tissue concentrations of Na^+^ (*p <* 0.01, *CV* = 16) and K^+^ (*p <* 0.05, *CV* =11) between the aneuploids and Chinese Spring are indicated by an asterisk (*).

Analysis of tissue Na^+^ concentration showed no difference between aneuploid lines and Chinese Spring in roots, bulked leaf blades or youngest fully expanded leaf blade regardless of low (0.2 mM) or high (4 mM) K^+^ status when genotypes were treated with an external solution containing 200 mM NaCl (Figure 6A). An increase of 24% in sheath Na^+^ concentration was observed in the aneuploids, 7AL-1 (Figure 6A) compared to Chinese Spring under both low and high K^+^ conditions. Similarly, Dt7BS showed 25% increase in sheath Na^+^ but only under low K^+^ conditions. Therefore, the chromosomal regions containing *TaHKT2;1 7AL-1* and *TaHKT2;1 7BL-1/-2* may have a significant effect on Na^+^ concentration in the sheath but not in the roots, bulk leaves nor the youngest fully expanded leaf blade. Interestingly, 7DL-2 null for *TaHKT2;1 7DL-*1 showed no significant difference between Chinese Spring (Figure 6A) indicating this gene does not have a role in controlling Na^+^ concentration in any tissue or treatment.

The same aneuploid lines when exposed to an external solution containing 200 mM NaCl and 4 mM K^+^ showed no differences in K^+^ concentration in bulked leaf blades regardless of the genotype or the Na^+^ status (Figure 6B). However, K^+^ concentration was reduced by 23% in the roots and 19% in the youngest fully expanded leaf blade for Dt7BS compared to Chinese Spring, but only under 200 mM NaCl treatment (Figure 6B). Similarly, sheath K^+^ was reduced by 12% in the aneuploid line 7AL-1 in the 200 mM NaCl treatment (Figure 6B). Therefore, it appears that the chromosomal regions containing *TaHKT2;1 7AL-1* and *TaHKT2;1 7BL-1/-2* influence K^+^ status under high NaCl conditions in a tissue-specific manner. There was no significant difference in K^+^ between 7DL-2 and Chinese Spring (Figure 6B) indicating that *TaHKT2;1 7DL-1* does not have a role in regulating K^+^ status in any tissue and treatments.

## Discussion

This study provides a detailed characterization of the *TaHKT2;1* multigene family in bread wheat which included four functional members and two pseudogenes. Despite presence of salt induced stress responsive elements in the promoter region it was found that the functional members of the *TaHKT2;1* family were expressed under low and high NaCl levels in roots and sheaths, but were down regulated in leaf blades. Wheat aneuploid lines were used to provide evidence for a potential role of individual *TaHKT2;1* gene family members in Na^+^ and K^+^ homeostasis in seedlings under low and high NaCl hydroponic conditions.

Although all members of the *TaHKT2;1* gene family contained three exons and two introns sharing the same gene structure as Group II *HKT* genes from other monocots [33], DNA sequence differences resulted in a degree of variability at the protein level. Variation amongst the *TaHKT2;1* genes and their encoded proteins could be of functional relevance. *TaHKT2;1* proteins potentially acting as K^+^/Na^+^ cotransporters have a conserved Gly-Gly-Gly-Gly signature in the selectivity filter, yet the filter signature alone does not necessarily predict ion selective properties and other domains responsible for ion specificity need to be considered [34,35]. In this regard, induced mutations within the cytoplasmic (L149, E180) or P-loop (A240, L247, F463 and E464) domains affected selectivity and the rate of uptake of K^+^ and Na^+^ by the *TaHKT2;1 7BL-1/-2* proteins in yeast and/or *Xenopus* oocytes heterologous systems [17,19,21,23,36,37]. Interestingly, the majority of the amino acids that were mutated in previous experiments were conserved between the members of *TaHKT2;1* family except for a A240S substitution in *TaHKT2;1 7DL-1*. In addition to A240S, five amino acid differences were identified within six base pair proximity to the filter glycine residues in the P-loops of *TaHKT2;1 7AL-1, TaHKT2;1 7DL-1* compared to *TaHKT2;1 7BL-1/-2*. Therefore, it is reasonable to hypothesize that the amino acid differences between members of the *TaHKT2;1* gene family in these regions may affect ion uptake properties (e.g. specificity and rate of ion transport). Since this study cloned FL-cDNAs of each member of the gene family, further investigations can be pursued using heterologous systems to evaluate the functional significance of the protein sequence variability for ion transport in the *TaHKT2;1* gene family.

*TaHKT2;1 7AL-2 *and *TaHKT2;1 7AL-3* shared typical characteristics of unprocessed pseudogenes, such as in-frame stop codons, location within close proximity to the functional genes on the same chromosome, and the absence of transcription [38]. Pseudogenes have previously been reported in the *HKT* super-family but their functional relevance is unknown [39]. Pseudogenes are thought to be an integral feature in evolution of polyploidy genomes, representing 12% of the genes in bread wheat [40] and may be of importance in gene or genome function. Pseudogenes in eukaryotes have been reported to have a regulatory role for genes from which they were derived, have non-coding functions or act as a source of genetic variability and were subjected to natural selection [38]. Conservation across progenitor genomes indicated that the single base deletion in exon 1 that caused a pre-mature stop codon in *TaHKT2;1 7AL-2* and *TaHKT2;1 7AL-3* was an ancient event created during evolution of the A genome progenitor species prior to, and maintained during, polyploidization of the tetraploid and hexaploid genomes. The *Ka/Ks* between the functional genes was close to the average *Ka/Ks* ratio of 0.2 previously estimated for functional wheat genes [41], providing evidence that the functional members of the *TaHKT2;1* gene family was subjected to resilient purifying selection. Although *TaHKT2;1 7AL-2* and *TaHKT2;1 7AL-3* had characteristics of pseudogenes the *Ka/Ks* value between these genes and *TaHKT2;1 7AL-1* was less than the theoretical value of 1.0 for a bona-fide pseudogene [38], indicating a relaxed selective constraint during genome evolution. Furthermore, the fact that these pseudogenes were retained during evolution of the hexaploid wheat genome with gene structure conservation could indicate some functional importance.

Each member of the *TaHKT2;1* gene family was variable for presence and frequency of salt induced CREs. Plant response to salinity is mediated by immediate and extensive reprogramming of the spatial and temporal gene transcription, instigated by the interaction of transcription factors with four major groups of CREs including ABA-independent CREs (CBF/DREB; NAC and ZF-HD) and ABA-dependent CREs (AREB/ABF; MYC/MYB) [42,43], all of which were represented in *TaHKT2;1* gene family. Interestingly, promoter diversity in *AtHKT1;1* genes resulted in variable salt response in natural populations of *Arabidopsis*[44]. Similarly, variability in CREs contributed to diverse expression patterns of *TaHKT1;5* in bread wheat and wild relatives under salt stress [45], supporting the hypothesis that *HKT* genes are regulated by CREs mediated mechanisms. The presence of these elements with varying copies and combinations predict salt response with variable expression patterns of individual *TaHKT2;1* members.

Given the CREs identified in this study, it was expected that each *TaHKT2;1* gene would show diverse transcriptional regulation, yet, no obvious transcript differences were detected between low and high NaCl treated plants. Similar observations were reported [24] where saline treatments imposed with K^+^ deficient conditions did not induce differences in *TaHKT2;1* gene expression in two week old wheat seedlings. However, expression of *TaHKT2;1 7BL-1/-2* was up regulated by imposing K^+^ starved conditions in six day old wheat [46] indicating a rapid response of genes to external K^+^ in younger seedlings. In the present study, there was an obvious down regulation of *TaHKT2;1* transcripts in leaf blades regardless of the NaCl concentration of the external solution, confirming adjustment of gene expression in this tissue. The extent of gene expression was further explored by mining wheat EST databases including NCBI, Transcriptome Shotgun Assembly and the TIGR Plant Transcript Assemblies, identifying a number of ESTs derived from pre-anthesis spikes and kernel tissue with 98-100% identity to members of the *TaHKT2;1* gene family (data not shown). Further studies are needed to understand the expression of *TaHKT2;1* gene family and how the various members influence ion homeostasis during different stages of plant development, various environmental conditions and variable salt levels.

An interesting feature of *TaHKT2;1* gene expression was the level of complexity caused by alternative splicing of a 571 bp transcript unique to *TaHKT2;1 7AL-1*. Alternative splicing is evident in 25 to 42% of bread wheat genes whereby some forms are thought to play a role in regulating functional transcript levels [47]. Alternative splicing at non-conventional–splice junctions has been reported in rice *OsHKT1;5* resulting in a premature stop codon and non-functional transcripts as a possible means of regulating mRNA degradation [34]*.* Similarly, alternative splicing event in *TaHKT2;1 7AL-1* indicates a rare unstable splice-junction motif that may act as an embedded molecular switch for post-transcriptional regulation and mRNA turn-over processes, which presumably may then influence levels of protein production.

The use of wheat aneuploid lines null for specific members of the *TaHKT2;1* family provided a system to analyse phenotypic effects of the genes on tissue ion concentrations. This approach provided evidence that a gene does not have a specific role when there was no significant difference between its corresponding aneuploid line and Chinese Spring (containing the full chromosome complement) whereas differences indicated that the gene would be a candidate controlling tissue Na^+^ and/or K^+^ status. The results, therefore, corroborate that *TaHKT2;1* genes neither regulate root Na^+^ uptake nor Na^+^ accumulation in bulk leaf blades and youngest fully expanded leaf blade. Nevertheless, presence of transcripts for all *TaHKT2;1* gene family members in these tissues under low and high NaCl conditions indicate alternative roles if they are translated into proteins. The higher sheath Na^+^ concentration in 7AL-1 and Dt7BS is likely from increased Na^+^ concentration in the xylem flow and being preferentially stored in sheaths. Wheat and other grasses do have mechanisms for withdrawal of Na^+^ from xylem in roots and sheath to reduce delivery of Na^+^ into the leaf blade [48]. Therefore, *TaHKT2;1 7AL-1* and *TaHKT2;1 7BL-1/-2* genes are candidates regulating Na^+^ accumulation in sheaths, perhaps by modifying Na^+^ in the root xylem that affects the flow of Na^+^ into sheaths. The possibility of other genes in the same chromosomal region contributing to this phenotypic effect cannot be excluded and further studies are needed to confirm the function of *TaHKT2;1* genes on 7AL and 7BL in controlling Na^+^ accumulation in the sheaths.

The *TaHKT2;1 7AL-1* gene may also regulate K^+^ accumulation in the sheath but only under high NaCl conditions, whereas *TaHKT2;1 7BL-1/-2* may serve a similar role in roots and the youngest fully expanded leaf blade, evident by the lower K^+^ in these tissues in the Dt7BS line. Since *TaHKT2;1* genes are not involved in K^+^ uptake by roots under saline conditions [18,49], the lower root K^+^ concentration observed for Dt7BS in this study would be due to a greater net K^+^ translocation from roots to shoots. Therefore, it is reasonable to assume that *TaHKT2;1 7BL-1/ TaHKT2;1 7BL-2* genes control K^+^ translocation in specific tissues of salt stressed wheat. Extensive functional redundancy of K^+^ transport systems [50] can be a challenge in isolated studies on individual transporter proteins, yet evidence in the present study identified the importance of further investigating *TaHKT2;1 7BL-1/TaHKT2;1 7BL-2 *and *TaHKT2;1 7AL-1* genes in controlling K^+^ homeostasis.

## Conclusions

*TaHKT2;1* genes has been of interest because the encoded proteins implement pathways for Na^+^ entry into wheat in saline conditions [24]. However, previous studies do not recognize a role of individual members of the *TaHKT2;1* gene family in controlling Na^+^ or indeed, of K^+^, during plant growth and development. The present study has highlighted diversity amongst the *TaHKT2;1* gene family and predicted proteins, as well as promoter variability in salt signalling and presence of *TaHKT2;1* transcripts in different tissues, under low and high levels of external Na^+^ and K^+^, gaining knowledge that *TaHKT2;1* genes could assume multiple roles in bread wheat. The phenotypic effects when individual genes were deleted in bread wheat showed that *TaHKT2;1* gene family are not involved in regulating root or leaf blade Na^+^ concentration but *TaHKT2;1 7AL-1* and *TaHKT2;1 7BL-1/-2* may be candidate genes controlling sheath Na^+^ concentration and K^+^ status in different tissues and experimental conditions. *TaHKT2;1 7DL-1* had no apparent role in regulating Na^+^ and K^+^ status in different tissue and treatments in this study. In summary, the present findings from combined molecular and physiological studies provided a broader perspective of the role of the *TaHKT2;1* family of genes in bread wheat.

## Methods

### Plant material

Bread wheat variety *Triticum aestivum* L. cv. Chinese Spring, six nullisomic-tetrasomic lines (N7AT7B, N7AT7D, N7BT7A, N7BT7D, N7DT7A, N7DT7B), eight deletion lines (7AL-1, 7AS-8, 7BL-2, 7BL-7, 7DL-2, 7DL-3, 7DL-5, 7DS-5) and ditelosomic lines (Dt7BL and Dt7BS) were kindly provided by Dr John Raupp, Wheat Genetic and Genomic Resources Centre, Kansas State University, USA. Six hexaploid cultivated bread wheat varieties Calingiri, Ducula, Krichauff, Westonia, SARC-1 and a tetraploid variety (*T. turgidum* L.) Tamaroi were obtained from the germplasm collection at the Department of Agriculture and Food, Western Australia. DNA samples of the diploid species, *T. monococcum* (accession 4044) was provided by Dr Evans Lagudah, CSIRO Plant Industry, Canberra, Australia.

### *In-silico* analysis of *TaHKT2;1* gene family

The *TaHKT2;1* FL-cDNA, U16709, was used as the query sequence in BLASTN to search IWGSS database (http://www.wheatgenome.org) and retrieve coding and promoter regions of the individual members of *TaHKT2;1* gene family. *In-silico* gene structure predictions for individual members of the *TaHKT2;1* family were made using Spidey (http://www.ncbi.nlm.nih.gov/spidey/), GeneSeqer (http://www.plantgdb.org/cgi-bin/GeneSeqer/index.cg) and SIM4 (http://pbil.univ-lyon1.fr/sim4.php) software. Intron-exon structures for each member were confirmed by aligning retrieved genomic sequences with the FL-cDNA, U16709. DNA and protein sequence analysis and structure predictions were made using GENEIOUS 6.0.3 [51] and phylogenetic analysis was performed in MEGA 5.05 [52]*.*

The predicted proteins were analysed for structural and functional elements including hydrophobicity and membrane topology using TMHMM Server 2.0 (http://www.cbs.dtu.dk/services/TMHMM/) and MPEx 3.2 [53] (http://blanco.biomol.uci.edu/mpex/) software suites. Three dimensional structures of the proteins were predicted by PHYRE 2 (http://www.sbg.bio.ic.ac.uk/phyre2/html/page.cgi?id=index). Protein sequence alignments, hydrophobicity plots and the 3-D structures were used to elucidate structural domains of the putative proteins.

DNA sequence 2000 bp upstream of predicted initiation site were analysed for *cis*-acting regulatory elements (CREs) associated with salt stress response in PlantCARE [54] and PLACE [55] databases.

### DNA extraction and PCR amplification

Plant genomic DNA was extracted from leaves using a phenol-chloroform-based method as described in [56] and was used as a template for PCR to amplify individual members of the *TaHKT2;1* family. Gene specific primer pairs were designed by targeting insertions and/or deletions (INDELs) and single nucleotide polymorphisms (SNPs) based on 3′ terminus mismatch described in [57]. NetPrimer (http://www.premierbiosoft.com/netprimer/netprlaunch/netprlaunch.html) was used to confirm primer compatibility. Primer information is summarized in Table 1. PCR reactions contained 1.5 mM MgCl_2_, 0.2 mM of each deoxyribonucleotides, 10 μM of each primer, 1X PCR buffer, 0.1 U *taq* DNA polymerase (BIOTAQ™ DNA Polymerase, Bioline, Australia) and 50 ng of template DNA in a 20 μl reaction volume. Cycle conditions for standard PCR were 35 cycles of 94°C 30 sec, primer annealing temperature 30 sec, 72°C 30 sec and the final extension 7 min at 72°C; and for touchdown PCR were five cycles of 94°C 30 sec, 55-50°C 30 sec, 72°C 30 sec; and then 35 cycles of 94°C 30 sec, 50°C 30 sec, 72°C 30 sec and the final extension 7 min at 72°C. PCR products were separated on 1.5% agarose gel in 0.5X Tris-acetate EDTA at constant voltage (90 V) for 30 min and visualized under UV light using Gel Doc System (BioRad, Italy) after staining with ethidium bromide.

### Hydroponic screening and tissue Na^+^ and K^+^ analysis of wheat aneuploid lines null for *TaHKT2;1* genes

Seeds of six selected wheat deletion and ditelosomic lines were washed in 0.04% sodium hypochlorite (42 g/L) for 30 sec, rinsed in deionized water, and placed on a mesh float on aerated 0.1 strength nutrient solution in the dark for three days. Seedlings were then transferred to 0.25 strength nutrient solution for one day. Nutrient composition at full strength was: (mM) K^+^, 3.95; Ca^2+^, 4; Mg^2+^, 0.4; NH_4_^+^, 0.625; NO_3_^−^, 4.375; SO_4_^2−^, 1.9; HPO_4_^2−^, 0.2; Fe-EDTA, 0.05; MES, 1.0; and micronutrients of one-quarter-concentration in Hoagland solution (pH was adjusted to 6.5 using Ca(OH)_2_). Four day old seedlings were then transferred to full strength nutrient solution in aerated, foil-covered, 4.5 L pots for 12 days. Each pot carried one individual of each of all six genotypes arranged randomly in each pot. There were four replicates of each genotype for each treatment in the experiment and the pots were arranged in a completely randomised design. Seedlings were held upright by a foam holder at the stem base inserted into individual holes in the pot lids. The experiment was carried out in a temperature controlled phytotron (20 ± 3°C/15 ± 2°C day/night) and the photosynthetically active radiation (PAR) recorded at midday was at ca. 1,400 - 1,500 μmol m^−2^ s^−1^. Solution levels in pots were maintained by topping up with deionized water.

In order to induce expression and study phenotypic effects of the *TaHKT2;1* gene family, the plants were subjected to low K^+^ conditions prior to imposing salt treatments [24,46]. As known *TaHKT2;1* transporters might be involved with either Na^+^ and/or K^+^ transport [16,21,22] NaCl treatments were also imposed at the two K^+^ levels: low (0.2 mM) K^+^, where high affinity transport systems for K^+^ dominate and high (4 mM) K^+^, where K^+^ uptake kinetics was determined by low affinity systems [58]. 12 day old seedlings were subjected to low K^+^ conditions for four days in a modified nutrient solution without added K^+^. The media contained (mM) Ca^2+^, 4; Mg^2+^, 0.4; NH_4_^+^, 0.825; NO_3_^−^, 4.375; SO_4_^2^- , 0.4; HPO_4_^2^-, 0.2; Fe-EDTA, 0.05; MES, 1.0; and micronutrients at one-quarter-concentrations in Hoagland solution (pH = 6.5, adjusted with Ca(OH)_2_) at a background Na^+^ and K^+^ concentrations of 0. 3 mM and 0.1 mM, respectively. After 4 days of low K^+^ level pre-treatment, 200 mM NaCl was applied to designated pots, so as to obtain four different Na^+^/K^+^ combinations: no added Na^+^/0.2 mM K^+^, no added Na^+^/4 mM K^+^, 200 mM Na^+^/0.2 mM K^+^ and 200 mM Na^+^/4 mM K^+^. The NaCl treatment was applied in 50 mM increments at 12 hour intervals and plants were maintained at the final 200 mM concentration for a full 3 days. The youngest fully expanded leaf blade, bulk leaf blades (all others), sheaths and roots of each plant were sampled. Prior to excision, roots were washed three times for 10 secs each in a solution containing 4 mM CaSO_4_ and 368 mM mannitol (200 mM treated plants) or in 4 mM CaSO_4_ (plants subjected to no added NaCl). Leaves and sheath samples were rinsed in deionized water. All tissue samples for ion analyses were oven-dried at 70°C, weighed and then ground to a powder. Samples of dried powdered tissue (100 mg) were extracted in 5 ml of 0.5 M HNO_3_ for 3 days on a mechanical shaker. Na^+^ and K^+^ were measured in technical triplicate using a flame photometer (Sherwood 410, Cambridge) and mean values represented each treatment replicate tissue sample. The reliability of the methods was confirmed by analyses of a reference tissue (broccoli, ASPAC Plant number 85) taken through the same procedures. Mean values from 4 treatment replicates of each genotype were analysed by ANOVA using SAS 9.4 (SAS Inc., USA) software.

### RNA extraction and RT-PCR

*T. aestivum* var. Chinese Spring was grown in hydroponics and treated with NaCl as described above. Leaf blades (including youngest fully expanded leaf blade), sheaths and roots of plants treated with 4 mM K^+^ with no added Na^+^ and 4 mM K^+^ with 200 mM NaCl were harvested after treating the plants for three days, snap frozen in liquid nitrogen for RNA extraction and RT-PCR. Duplicate tissue samples were harvested for Na^+^ analysis (described above). Total RNA was isolated using RNeasy Plant Mini Kit (Qiagen, Hilden, Germany). First strand cDNA was synthesised using RT Omniscript Kit (Qiagen, Hilden, Germany). Integrity of synthesised cDNA was verified by RT-PCR using primers (forward 5′-CGCCAGGGTTTTCCCAGTCACGAC −3′, reverse 5′-TCACACAGGAAACAGCTATGAC −3′) designed for glyceraldehyde-3-phosphate dehydrogenase (GAPDH) gene [59]. FL- cDNA of individual members of the *TaHKT2;1* gene family were amplified from cDNA preparations using primer pairs FL:A1 F and FL:A1 R, FL:A2/3 F and FL:A2/3R, FL:B1/2 F and FL:B1/2 R and FL:D1 F and FL:D1 R (Table 1).

### FL-cDNA cloning and sequencing

RT-PCR amplicons were separated and excised from 1% agarose gels, purified using Wizard® SV Gel and PCR Clean-Up System (Promega, CA,USA), and cloned into pGEM®-T Easy Vector Systems (Promega, CA, USA). Three bacterial colonies containing cloned *TaHKT2;1* FL-cDNA were selected and DNA templates were purified using Wizard® Plus SV Minipreps (Promega, CA, USA). The cloned fragments were sequenced using BigDye™ sequencing chemistry (Applied Biosystems, Perkin Elmer, Weiterstadt, Germany) and analysed using GENEIOUS 6.0.3 [51].

## Competing interests

The authors declare they have no competing interests.

## Authors’ contributions

HACKA conducted the bioinformatic work, designed the experiments, generated and analysed data, interpreted results and wrote the manuscript. TUH contributed to designing and conducting experiments for transcript analysis. TDC contributed to designing phenotyping experiments, data interpretation and edited the manuscript. MGF contributed to design of all experiments, data interpretation and edited the manuscript. All authors read, revised and approved the final manuscript.

## Supplementary Material

Additional file 1: Figure S1 Hydrophobicity of predicted proteins from the functional members of the *TaHKT2;1* gene family. Click here for file
