# Supplementary material for: Characterization of the multigene family TaHKT 2;1 in bread wheat and the role of gene members in plant Na+ and K+ status
Source: BMC Plant Biol. 2014 Jun 11;14:159. doi: 10.1186/1471-2229-14-159 (PMC4079177; doi:10.1186/1471-2229-14-159)
Supplement: Additional file 1: Figure S1 — Hydrophobicity of predicted proteins from the functional members of the TaHKT2;1 gene family. [file 1471-2229-14-159-S1.pptx]

## Slide 1
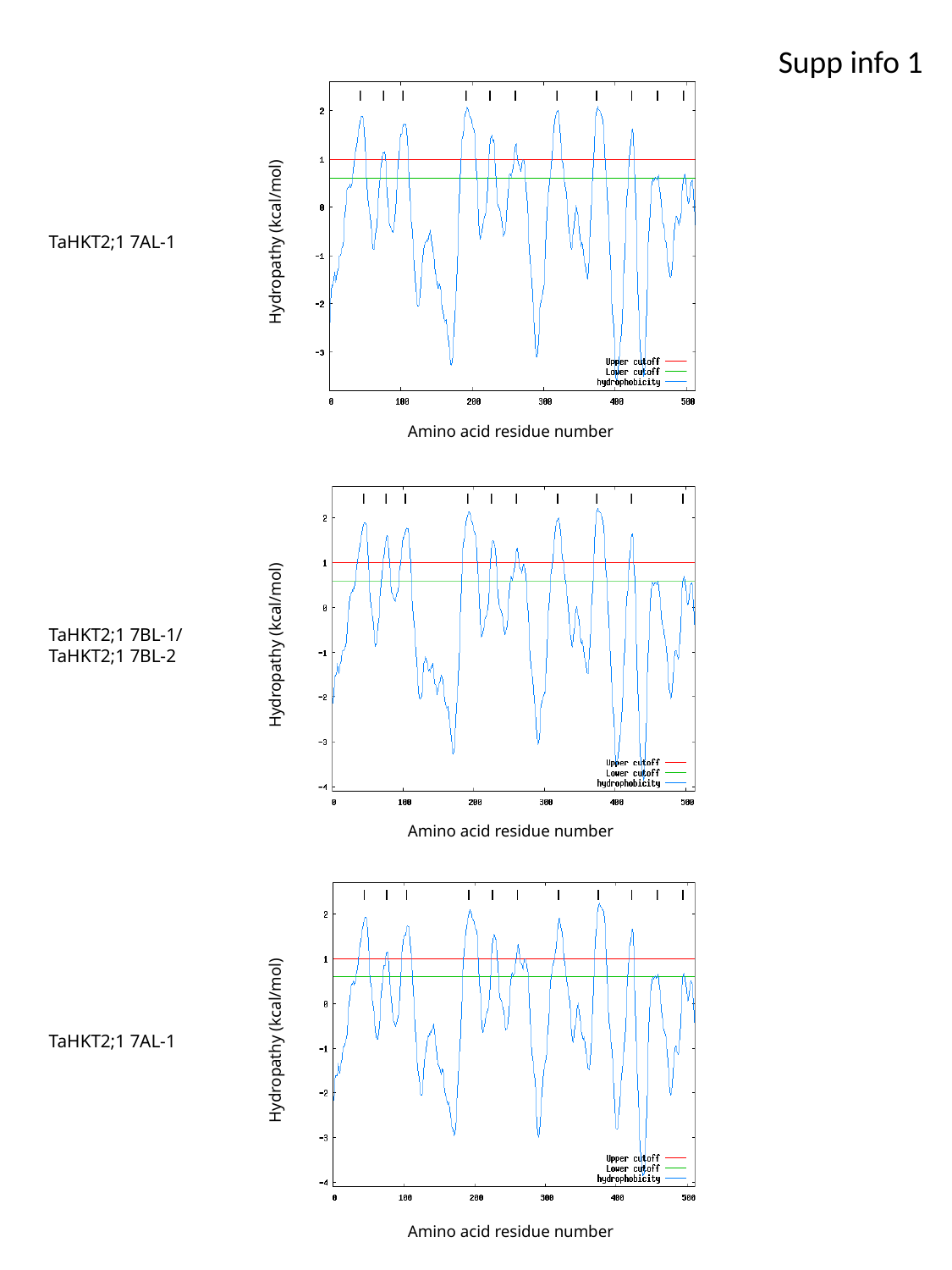

Supp info 1
Hydropathy (kcal/mol)
TaHKT2;1 7AL-1
Amino acid residue number
Hydropathy (kcal/mol)
TaHKT2;1 7BL-1/ TaHKT2;1 7BL-2
Amino acid residue number
Hydropathy (kcal/mol)
TaHKT2;1 7AL-1
Amino acid residue number
